# Editorial: Bridging Gaps Between Sex and Gender in Neurosciences

**DOI:** 10.3389/fnins.2020.00561

**Published:** 2020-06-11

**Authors:** Annie Duchesne, Belinda Pletzer, Marina A. Pavlova, Meng-Chuan Lai, Gillian Einstein

**Affiliations:** ^1^Department of Psychology, University of Northern British Columbia, Prince George, BC, Canada; ^2^Department of Psychology, University of Salzburg, Salzburg, Austria; ^3^Centre for Cognitive Neuroscience, University of Salzburg, Salzburg, Austria; ^4^Department of Psychiatry and Psychotherapy, Medical School and University Hospital, Eberhard Karls University of Tübingen, Tübingen, Germany; ^5^Margaret and Wallace McCain Centre for Child, Youth & Family Mental Health, Azrieli Adult Neurodevelopmental Centre, and Campbell Family Mental Health Research Institute, Centre for Addiction and Mental Health, Toronto, ON, Canada; ^6^Department of Psychiatry and Autism Research Unit, The Hospital for Sick Children, Toronto, ON, Canada; ^7^Department of Psychiatry, Faculty of Medicine, University of Toronto, Toronto, ON, Canada; ^8^Department of Psychiatry, University of Cambridge, Cambridge, United Kingdom; ^9^Department of Psychiatry, National Taiwan University Hospital and College of Medicine, Taipei, Taiwan; ^10^Department of Psychology, University of Toronto, Toronto, ON, Canada; ^11^Rotman Research Institute, Baycrest Hospital, Toronto, ON, Canada; ^12^Department of Gender Studies, Linköping University, Linköping, Sweden; ^13^Canadian Consortium on Neurodegeneration and Aging, Toronto, ON, Canada; ^14^Wilfred and Joyce Posluns Chair in Women's Brain Health and Aging, Toronto, ON, Canada

**Keywords:** sex, gender, gonadal hormones, epigenetic, cognition, stress, pain, brain injury

Individual differences are shaped by a myriad of interrelated factors depending on how the nervous system develops, adapts, reacts to, and interacts with the world outer settings. Sex-related variables represent a set of sexually-defining biological characteristics including chromosomes, patterns of gene expression, hormone levels, and reproductive/sexual anatomy. These variables have been extensively linked to the development and functioning of the nervous system (Pletzer, 2015; Prager, 2017). The sex of an organism has been associated with a plethora of well-established (Jonasson, 2005; Mogil, 2012; Stevens and Hamann, 2012; Yagi and Galea, 2019) and controversial (Eliot, 2011) nervous system differences. However, sex-related biological variables rarely fully explain nervous system differences between male and female individuals (Eliot and Richardson, 2016), particularly in humans (Pavlova, 2017a; Rippon et al., 2017). In concert with biological differences, women and men differ in their experiences of the social world. Gender-related variables, including gendered behaviors, relations, expectations, beliefs, and attitudes that are experienced throughout the lifespan, have also been associated with differences in brain function and behavior across individuals (Einstein, 2007; Rippon et al., 2014; Jordan-Young et al., 2019). Sex- and gender-related variables dynamically influence our biology and the environment such that these variables are continuously shaping and being shaped in a reciprocal relationship with the world. As scientific and clinical communities recognize the need for neuroscientific inquiry that interrogates the combined contributions of sex- and gender-related variables, the call to action multiplies (Kimerling et al., 2018; Nebel et al., 2018; Grissom and Reyes, 2019; Tannenbaum et al., 2019). This special topics issue of Frontiers assembles a collection of research, reviews and commentaries that propose new approaches to the integration of sex- and gender-related variables in neuroscience.

The constructs of sex and gender as defined in this text are often not dissociable in the literature, especially when individuals are categorized as women or men. Categorizing individuals as “women and men” or “male and female” challenges our capacity to interrogate the relative contributions of gender-related factors to understanding individual differences. That said, even when using the broad category of “women and men,” novel analytical approaches can improve our characterization of where and when sex- and gender-related differences occur. Four articles within this Issue reveal how the binary category, “women and men,” continues to moderate brain and psychological processes. Luo et al. demonstrate that multivariate classification approaches with high-dimensional data (e.g., tens of thousands of features per subject/observation) from cortical brain morphology can categorize adult individuals as women or men, replicating previous findings conducted with high-dimensional, large sample size, and multivariate approaches (Chekroud et al., 2016; Rosenblatt, 2016; Anderson et al., 2019). In another study, Stam et al. demonstrate opposing associations between personality traits and gray matter brain volume in individuals grouped as women or men. Two articles provide novel insights into the relevance of the “women and men” as categories for understanding psychological processes. In a study exploring how individuals infer social signals from bodies and eyes of others, Isernia et al. report that categorizing participants as “women and men” reveals that individuals within each group may use different sources of information and perceptual strategies to achieve similar level of performance accuracy in social cognition tasks. Perchtold et al. first demonstrate that individuals categorized as women or men are equally able to generate cognitive reappraisals for anxiety-inducing situations, but that reappraisal ability is only predictive of reduced depressive symptoms in those categorized as men. These studies expand the knowledge foundation upon which elements of sex and gender can be further interrogated.

Improving our neuroscientific understanding of sex and gender can be achieved through direct pharmacological and physiological manipulation. For instance, Derntl et al. demonstrate that the dissociative experience (e.g., following the administration of a subclinical dose of ketamine) differs between individuals categorized as women or men. Similarly, Wang et al. show that the effects of mPFC transcranial direct current stimulation (tDCS) on implicit gender stereotype bias differs between individuals categorized as women or men. Addressing the larger issue of how the “women and men” category moderates the effects of pharmacological and physiological manipulations enhances the ability to make nuanced behavioral predictions and provides critical information for future clinical trial design.

Three studies in this Issue investigate the unique and relative contributions of both sex- and gender-related variables to brain and psychological processes. Hornung et al. preliminary findings demonstrate that the recruitment of brain regions during the processing of gendered self attributes varies according to circulating levels of sex hormones in individuals categorized as women or men. Plezter et al. re-examine the previously-reported finding that the “women and men” category is a reliable predictor of spatial ability and reveal that this association disappears when accounting for the interactive effects of circulating levels of gonadal hormones and self-reported endorsement of stereotypical attitudes and activities. Adopting a similar analytical approach in another study, Plezter interrogates the interaction between gonadal hormones and gendered attitudes and activities in predicting grey matter volume (Pletzer). These studies highlight how important it is to go beyond “women and men” as categories within the realm of neuroscientific inquiry, as they may be obscuring relationships that can be better explained through more nuanced biosocial interactionist approaches.

The prevalence of a number of clinical conditions differs as a function of the “women and men” categories. While an increasing number of theoretical models explore these differences by integrating dimensions of sex- and gender-related variables (Lai et al., 2015; Becker et al., 2017; Nebel et al., 2018), most studies tend to restrict causal explanations to either sex- *or* gender- related variables (Li and Graham, 2017; Hillerer et al., 2019; Slavich and Sacher, 2019). Thus, looking at interaction between sex- and gender-related variables in clinical conditions may have theoretical and therapeutic benefits. In a critical review of the literature on fibromyalgia, Meester et al. propose a model integrating sex- and gender-related variables. Investigating physiological correlates of consciousness in patients with traumatic brain injury, Zhong et al. demonstrate that high circulating levels of testosterone within a week following the trauma predicted regaining of consciousness only in individuals identified as men. Recognizing and integrating sex- and gender-related variables is central for furthering our understanding of the brain and moving toward the development of personalized precision medicine.

Measurements, tasks, tests, and experimental manipulations are developed and validated under a number of assumptions that often do not account for the possible roles of sex- and gender-related variables. Re-examining and validating methodologies across sexes and genders is a crucial step; when validation has not been conducted with sex- and gender-related differences in mind, discriminating between true differences and methodological artifacts is simply impossible (McCarthy et al., 2017; Rich-Edwards et al., 2018). For instance, in a critical review of individual differences in placebo/nocebo effects, Enck and Klosterhalfen report that differences observed between women and men are more commonly reported in experimental studies than in randomized clinical trials, suggesting that methodological bias may contribute to apparent systemic group-level differences. Building on past stress paradigms, Tops et al. developed and validated a new neuroimaging virtual social rejection stress paradigm reproducing peer exclusion commonly experienced on social media platforms and allowing for a more specific investigation of possible sex- and gender-related differences in the neurophysiological processes of peer social rejection. Finally, Jones et al. reveal independent contributions of combined estradiol and testosterone to sexual behavior in female rats, demonstrating the empirical value of examining the role of multiple sex steroid hormones within all individuals. Revisiting and developing new methodologies that account for the possible contributions of sex- and gender-related variables is essential to provide a valid foundation of neuroscientific inquiry.

Ultimately, the research on sex and gender in neuroscience is constrained by issues with operationalizing definitions of sex and gender. Two articles in this Research Topic reconsider the stability of sex and gender as separate, uniform constructs, and how sex and gender relate to one another in the pursuit of understanding *individual* rather than category-based differences in neuroscience. Holmes and Monks argue that the very categories of sex and gender are problematic in attempting to bridge these constructs with neuroscientific questions. Similarly, Cortes et al. propose that, rather than treating sex and gender as discrete boxes, researchers should focus on understanding an individual's experiences of sex and gender as products of interactive, dynamic and multifaceted epigenetic processes. By focusing on individual-level variables rather than broad categories, these new conceptual frameworks facilitate the understanding of individual differences in neuroscientific processes.

Challenges remain for the bridging of sex and gender dimensions in neuroscience, and, in particular, in our understanding of the social brain (e.g., Pavlova, 2017a,b); some of these are apparent from the studies in this Issue. For example, the varied terminology employed in describing the often category-based sex- and gender-related differences across the different papers within this special issue highlights the need for researchers and clinicians to more consistently and explicitly operationalize their usage of these terms (Clayton and Tannenbaum, 2016). As well, most of the work this Issue operationalizes “women and men” as binary categories (Hyde et al., 2019), which is understandable considering how individuals are often categorized in research, but may in fact be of questionable validity considering our current state of understanding the multi-dimensional nature of sex- and gender-related variables (Johnson et al., 2009). Ultimately, the field will benefit most from going beyond the dichotomous categories of sex and gender and embracing interactionist models, as underscored by some of the papers in this special issue on Sex and Gender in Neuroscience.

## Author Contributions

AD conceptualized, drafted, and implemented the suggestions by co-authors. BP provided the significant practical and conceptual suggestions to the manuscript. MP provided the multiple practical and conceptual feedback suggestions to the manuscript and proofread the final version. M-CL provided the practical and conceptual suggestions to the manuscript. GE provided the practical and conceptual suggestions to the manuscript. All co-authors contributed to the writing of the paper.

## Conflict of Interest

The authors declare that the research was conducted in the absence of any commercial or financial relationships that could be construed as a potential conflict of interest.
